# Sex steroid levels in corresponding cerebrospinal fluid and serum samples quantified by mass spectrometry in men

**DOI:** 10.1530/EC-23-0250

**Published:** 2023-12-14

**Authors:** Henrik Ryberg, Anna-Karin Norlén, Andreas Landin, Per Johansson, Zeinab Salman, Anders Wallin, Johan Svensson, Claes Ohlsson

**Affiliations:** 1 Department of Clinical Chemistry, Sahlgrenska University Hospital, Gothenburg, Sweden; 2 Sahlgrenska Osteoporosis Centre, Center for Bone and Arthritis Research (CBAR), Department of Internal Medicine and Clinical Nutrition, Institute of Medicine, University of Gothenburg, Gothenburg, Sweden; 3 Department of Psychiatry and Neurochemistry, Institute of Neuroscience and Physiology, Sahlgrenska Academy, University of Gothenburg, Mölndal, Sweden; 4 Department of Endocrinology, Skaraborg Central Hospital, Skövde, Sweden

**Keywords:** neuroendocrinology, androgen, estrogen, mass spectrometry

## Abstract

**Objective:**

Sex steroids exert important biological functions within the CNS, but the underlying mechanisms are poorly understood. The contribution of circulating sex steroids to the levels in CNS tissue and cerebrospinal fluid (CSF) has been sparsely investigated in human and with inconclusive results. This could partly be due to lack of sensitive validated assays. To address this, we validated a gas chromatography–tandem mass spectrometry (GC-MS/MS) assay for quantification of sex steroid hormones/precursors in CSF.

**Methods:**

GC-MS/MS quantification of dihydrotestosterone (DHT, CSF lower limit of quantification, 1.5 pg/mL), testosterone (4.9), estrone (E1, 0.88), estradiol (E2, 0.25), dehydroepiandrosterone (DHEA, 38.4), androstenedione (4D, 22.3), and progesterone (P, 4.2) in CSF, and corresponding serum samples from 47 men.

**Results:**

Analyses of CSF revealed that DHEA was the major sex steroid (73.5 ± 31.7 pg/mL) followed by 4D (61.4 ± 29.6 pg/mL) and testosterone (49.5 ± 18.9 pg/mL). The CSF levels of DHT, E2, and E1 were substantially lower, and P was in general not detectable in CSF. For all sex steroids except E2, strong associations between corresponding CSF and serum levels were observed. We propose that testosteronein CSF is derived from circulating testosterone, DHT in CSF is from local conversion from testosterone, while E2 in CSF is from local conversion from 4D in CNS.

**Conclusions:**

We describe the first thoroughly validated highly sensitive mass spectrometric assay for a broad sex steroid hormone panel suitable for human CSF. This assay constitutes a new tool for investigation of the role of sex steroid hormones in the human CNS.

**Significance statement:**

In this study, a fully validated highly sensitive mass spectrometric assay for sex steroids was applied to human CSF. The results were used to describe the relative contribution of peripheral circulating sex steroids together with locally transformation of sex steroids to the levels in CSF. The results are of importance to understand the biological processes of the human brain.

## Introduction

Sex steroid hormones are predominantly produced from cholesterol by the gonads and the adrenal glands. Then, the sex steroids are distributed to target tissues through the circulation. In addition, many tissues and organs, including the CNS, possess the ability of converting cholesterol into bioactive steroids. For example, astrocytes and neurons can convert cholesterol into testosterone, estrone (E1), estradiol (E2), dehydroepiandrosterone (DHEA), androstenedione (4D), and progesterone (P) (1).

Circulating sex steroid levels may contribute to the levels seen in cerebrospinal fluid (CSF), but the relative contributions of local production vs peripheral sources to sex steroid levels in the CNS are not well understood (2).

The low polarity of sex steroids enables the non-protein-bound fraction to readily pass through cell membranes, allowing them to pass from the circulation into CSF and brain tissue (3). In addition to the unconjugated sex steroids measured in this study, sulfated forms of the precursors DHEA and pregnenolone may also enter the CNS and potentially contribute to the CNS levels of sex steroid hormones (4). Locally in the brain, sex steroid hormones/precursors with weak bioactivities can be converted into more potent sex steroids. The steroidogenic machinery in the brain can produce pregnenolone directly from cholesterol, which can be further metabolized into different steroid hormones (5). For example, the sex steroid precursor DHEA can be converted by 3β-hydroxysteroid dehydrogenase isoenzymes into 4D, which can be further metabolized via 17β-hydroxysteroid dehydrogenase isoenzymes to the potent androgen testosterone. Furthermore, 4D and testosterone can be converted by aromatase to the estrogens E1 and E2. In addition, testosterone can be metabolized to the highly potent androgen DHT by 5α-reductase (1, 5). However, a more comprehensive description of the complex metabolism of steroids in the brain can be found elsewhere (6).

Sex steroids exert effects on neuronal cells such as stimulating neurogenesis and brain plasticity, thereby modulating CNS functions (6, 7). Furthermore, androgen and estrogen receptors, which mediate the effects of androgens (DHT and testosterone) and estrogens (E2 and E1), are widely expressed in the brain, e.g. in the cerebral cortex, hippocampus, cerebellum, and spinal cord (6, 8, 9, 10). However, the regulation of sex steroids levels in the CNS is poorly understood, which might be due to lack of sensitive and specific assays (11). In humans, sex steroid levels in CSF are estimated to be 10- to 100-fold lower compared to those in the circulation (12), and quantitation of these low sex steroid levels have many challenges. On the other hand, CSF contains lower levels of interfering substances such as proteins or excreted metabolites. Therefore, it can be assumed that assays validated for serum or urine could also be used for CSF measurements, given that they are sensitive enough to detect the low CSF levels and that complementary validations are made. Assays based on mass spectrometry (MS) offer superior combination of sensitivity and specificity and are therefore well suited for analysis of sex steroid hormones (11, 13). In human CSF, sex steroid hormone levels have only sparsely been investigated using MS, and only a few studies have presented assay-validation data including lower limits of quantifications (LLOQs), precisions and accuracies, rendering interpretation of earlier data difficult. To our knowledge, none of the previous studies have applied a fully validated, high-sensitive mass spectrometric assay for sex steroids on a large human cohort with corresponding CSF and serum samples.

Previously, we validated an assay using gas chromatography combined with tandem MS (GC-MS/MS) for measurements of sex steroids in analytically challenging matrices with low steroid levels such as serum and tissues from rodents as well as human serum (14, 15). Recently, we used this assay to describe the associations for CSF and serum levels of testosterone in men (16). In the present study, we adopted and validated this GC-MS/MS assay for high-sensitive measurements of a broad panel of sex steroid hormones/precursors (DHT, testosterone, E1, E2, DHEA, 4D, and P) in human CSF. This assay could, therefore, provide a new analytical tool to study the role of sex steroid hormones in the human brain. Here, we used this assay to describe associations between sex steroid levels in CSF and serum as well as the associations between different sex steroids in CSF. Finally, we used the data to estimate the relative contribution of peripheral circulating sex steroids to the CSF levels, and possible pathways of transformation of sex steroids within the CNS.

## Materials and methods

### Reagents

All reagents used were of pro analysis grade or better. Testosterone was purchased from Fluka (Buchs, Germany). All other reference material and reagents were from Sigma-Aldrich. Isotope-labeled internal standards of DHT (dihydrotestosterone-2,3,4-^13^C_3_), testosterone (testosterone-2,3,4-^13^C_3_), E1 (estrone-2,3,4-^13^C_3_), E2 (estradiol-2,3,4-^13^C_3_), 4D (androstenedione-2,3,4-^13^C_3_), DHEA (dehydroepiandrosterone-2,2,3,4,4,6-d_6_), and P (progesterone-2,3,4-^13^C_3_) were used as internal standards, for detailed information on internal standards see Supplementary Table 4 (see the section on supplementary materials given at the end of this article).

### Calibrators

The calibrator stock solutions of each sex steroid were prepared in ethyl acetate. Next, the stock solutions were pooled to a calibrator standard solution and diluted in methanol. For a detailed description, see Supplementary Table 3. Calibrator stock and standard solutions were aliquoted and stored in 2 mL vials at −80°C. Between experiments, thawed vials were stored at 4°C up to 4 weeks. On each day of analysis, 7-point calibration curves, including 0, were prepared from the calibrator standard solutions by dilution in water (1:10). Calibration points (pg/mL) for serum measurements ranged from 2.25 to 3600 for DHT, from 11.25 to 18,000 for testosterone, from 0.9 to 1440 for E1 and for E2, from 5.6 to 9000 for 4D, from 45 to 36,000 for DHEA, and from 22.5 to 36,000 for P. For the CSF measurements, the calibration points ranged from 1.25 to 2000 for DHT, from 6.25 to 10 000 for testosterone, from 0.5 to 800 for E1 and for E2, from 3.1 to 5000 for 4D, from 25 to 20,000 for DHEA, and from 12.5 to 20,000 for P. Calibration was performed by determining the peak area ratio between the target analyte and the corresponding isotope-labeled internal standard.

### Study subjects and sample collection

In the fasted state, blood sampling and lumbar puncture were performed simultaneously in 47 healthy individuals from the Västra Götaland County in Sweden. All blood samples were collected in the fasted state between 08.00 and 10.00 h. After the serum was separated, it was transferred to polypropylene tubes. The lumbar puncture was performed in the L3–L4 or L4–L5 interspace; CSF was collected in a polypropylene tubes and centrifuged at 2 000 ***g*** for 10 min. The collected serum and CSF were stored in −80°C pending analyses. The study was approved by the Regional Ethics Committee in Gothenburg, and oral and written consent was obtained from all patients. The research was performed according to the Declaration of Helsinki.

### Sample preparation and GC-MS/MS analysis

Detailed information on the assay originally developed for serum and tissues measurements has been published previously (14, 15), and the validation of CSF testosterone has previously been described (16). For the measurements, 450 µL CSF or 250 µL serum plus 200 µL deionized water were used. After this, 50 µL internal standards and 500 µL of 0.5 M ammonium acetate were added to the samples. Concentrations used for each internal standard can be found in Supplementary Table 3. The steroids were then extracted using 1-chlorobutane and purified on Silica SPE columns (Supra-Clean SI-S, PerkinElmer).

Oximation of the keto groups was performed using triethylamine and pentafluorobenzylhydroxylamine hydrochloride. Esterfication was performed using pentafluorobenzoyl chloride. Prior analysis, samples were dried down and reconstituted in 100 µL isooctane. The equipment constituted of an Agilent 7890A GC, an Agilent 7693 autosampler, two 50% phenyl-methyl polysiloxane (DB-17HT) capillary columns (15 m × 0.25 mm internal diameter, 0.15 m film thickness), and an Agilent 7000 triple quadrupole mass spectrometer (Agilent). Analytes were detected with electron capture negative chemical ionization in multiple reaction monitoring (MRM) mode. Ammonia was used as reagent gas. The following transitions were used for the quantification: estradiol (E2), 660.2→596.2; estradiol-^13^C_3_, 663.1→599.2; estrone (E1), 464.2→400.2; estrone-^13^C_3,_ 467.1→403.2; testosterone, 677.2→496.2; testosterone-^13^C_3_, 680.2→499.2; dihydrotestosterone (DHT), 679.2→181.0; dihydrotestosterone-^13^C_3_, 682.2→181.0; progesterone (P), 489.2→459.3; progesterone-^13^C_3_, 492.2→462.3; androstenedione (4D), 461.2→431.2; androstenedione-^13^C_3_, 464.2→434.2; dehydroepiandrosterone (DHEA), 482.2→167.0; dehydroepiandrosterone-d_6_, 488.2→167.0. All peaks were automatically integrated using the MassHunter quantitative analysis Workstation Software (version 10.1) from Agilent.

### Quality control

For every batch analyzed, external quality controls were included that consisted of pooled human and rat sera with well-defined concentration ranges. Values below LLOQ were regarded as unmeasurable and were replaced with the LLOQ values in the further statistical analyses. Finally, if more than half of the measurements were under LLOQ, the group mean was denoted as <LLOQ-value.

### Validation of the assay for CSF measurements

CSF samples from elderly subjects with low levels of endogenous sex steroids were merged into two pools for the validation experiments and the endogenous levels were determined. From these pools, a set of samples for validation experiments was created through spiking with sex steroid levels that we believed to be relevant for validation purposes. The final set of samples consisted of seven different levels with five aliquots per level. For intra-assay precision, sensitivity, and accuracy calculations; suitable samples from this set were measured in the same batch. For the interassay precision validation, performed later, we used another pool of samples from elderly subjects and these samples were also spiked to achieve low and high levels. Sensitivity was determined as Lowest Level of Quantification levels (LLOQ; pg/mL), defined as the lowest level with signal-to-noise (S/N) ratio exceeding three times, precision (CV, coefficient of variation) values under 20% and an accuracy of 80–120%. Accuracies were determined at two different levels, calculated as (measured level − baseline level)/spiked level × 100. For the interassay precision; the levels in two pools of CSF were measured once at 8 different days during a period of 1 month.

In general, a CV less than 20% at lowest calibration point (LLOQ) together with interassay and intra-assay CV values less than 10% and an accuracy between 80 and 120% were regarded as acceptable for the assay validation.

### Statistical analysis

The statistical evaluations were performed using SPSS (version 28; IBM Corp.). Values are given as the mean ± s.d. or median and range, unless otherwise indicated. Correlations were examined using Pearson’s correlation coefficient. The independent associations for different candidate sex steroids in CSF/serum were estimated by combined linear regression models, which included the two most plausible candidates for each evaluated CSF steroid (the strongest candidate from CSF and the strongest candidate from serum). A two-sided *P*-value less than 0.05 was considered significant.

## Results

### Assay performance for CSF measurements

Representative examples of extracted ion chromatograms of target steroids in CSF are shown in Supplementary Fig. 1. In pooled CSF, a functional lower limit of quantification (LLOQ) was established for each sex steroid hormone, defined as the level when the signal-to-noise ratio (S/N) was exceeded 3 times, the CV was below 20%, and the accuracy was between 80 and 120%. The assay was overall highly sensitive for all investigated sex steroids/precursors and the LLOQs (pg/mL) were DHT, 1.5; testosterone, 4.9; E1, 0.88; E2, 0.25; DHEA, 38.4; 4D, 22.3; and P, 4.2 (Table 1). Furthermore, the intra-assay precisions at two different levels are shown in Table 1. In a low level pool close to the LLOQ:s, the intra-assay CV values ranged from 2.2% to 10.9%, while in a high level pool, the CV values were all below 3%. The interassay CV values were all below 10% at two different levels (Table 1). Accuracies at two different levels, calculated as (measured level − baseline level)/spiked level × 100, were for DHT; 100% (low level) and 106% (high level), for testosterone; 108% and 101%, for E1; 83% and 97%, for E2; 120% and 109%, for DHEA; 87% and 91%, for 4D; 104% and 104%, and for P; 113% and 105% (Table 2).

**Table 1:** Sensitivity and precision in human CSF for sex steroid hormones.

	DHT	Testosterone	E1	E2	DHEA	4D	P
Sensitivity LLOQ (pg/mL)	1.5	4.9	0.88	0.25	38.4	22.3	4.2
Precision							
Intra-assay							
Level 1	10.9% (1.49)	4.5% (4.9)	3.1% (1.0)	5.9% (0.36)	2.2% (41.7)	3.8% (24.3)	3.1% (4.2)
Level 2	2.3% (104)	0.2% (530)	0.7% (41.2)	1.1% (42.5)	1.6% (1015)	0.3% (312)	0.7% (1024)
Interassay							
Level 1	4.7% (77.6)	1.4% (384)	4.3% (29.5)	8.2% (32.1)	4.5% (821)	4.8% (205)	0.8% (713)
Level 2	4.1% (409)	3.6% (2044)	4.5% (156)	4.9% (170)	4.1% (4164)	3.4% (1033)	4.3% (3815)

Sensitivity: lowest level of quantification levels (LLOQ; pg/mL) was defined as the lowest level with signal-to-noise (S/N) ratio exceeding three times and precision (CV, coefficient of variation) values <20%. Five replicates for each level evaluated were used for the sensitivity determinations. Precision: for intra-assay precision, five samples at two different levels were measured in the same batch. For the interassay precision, the levels in two pools of CSF were measured once at 8 different days during a period of 1 month. Data within brackets are the levels at which the CV was measured expressed as pg/mL. Part of the validation data for testosterone have previously been published (16).

4D, androstenedione; DHEA, dehydroepiandrosterone; DHT, dihydrotestosterone; E1, estrone; E2, estradiol; P, progesterone.

**Table 2:** Accuracy in human CSF for sex steroid hormones.

		Spiked pg/mL	Baseline pg/mL	Measured pg/mL	Accuracy %
DHT	Low level	1.5	0	1.50	100
	High level	40	1.77	44.1	106
Testosterone	Low level	7.5	1.53	9.62	108
	High level	200	26.5	228	101
E1	Low level	0.6	0.82	1.31	83
	High level	16	2.42	17.9	97
E2	Low level	0.3	0	0.36	120
	High level	16	0	17.5	109
DHEA	Low level	7.5	38.4	44.9	87
	High level	400	91.8	456	91
4D	Low level	100	52.3	156	104
	High level	250	52.3	312	104
P	Low level	3.75	0	4.23	113
	High level	15	0	15.7	105

Accuracies were calculated as (measured level − baseline level)/spiked level × 100. Five replicates for each level evaluated were used for the accuracy determinations. Part of the validation data for testosterone have previously been published (16).

4D, androstenedione; DHEA, dehydroepiandrosterone; DHT, dihydrotestosterone; E1, estrone; E2, estradiol; P, progesterone.

### Sex steroid levels in human serum and CSF

**Figure 1:**
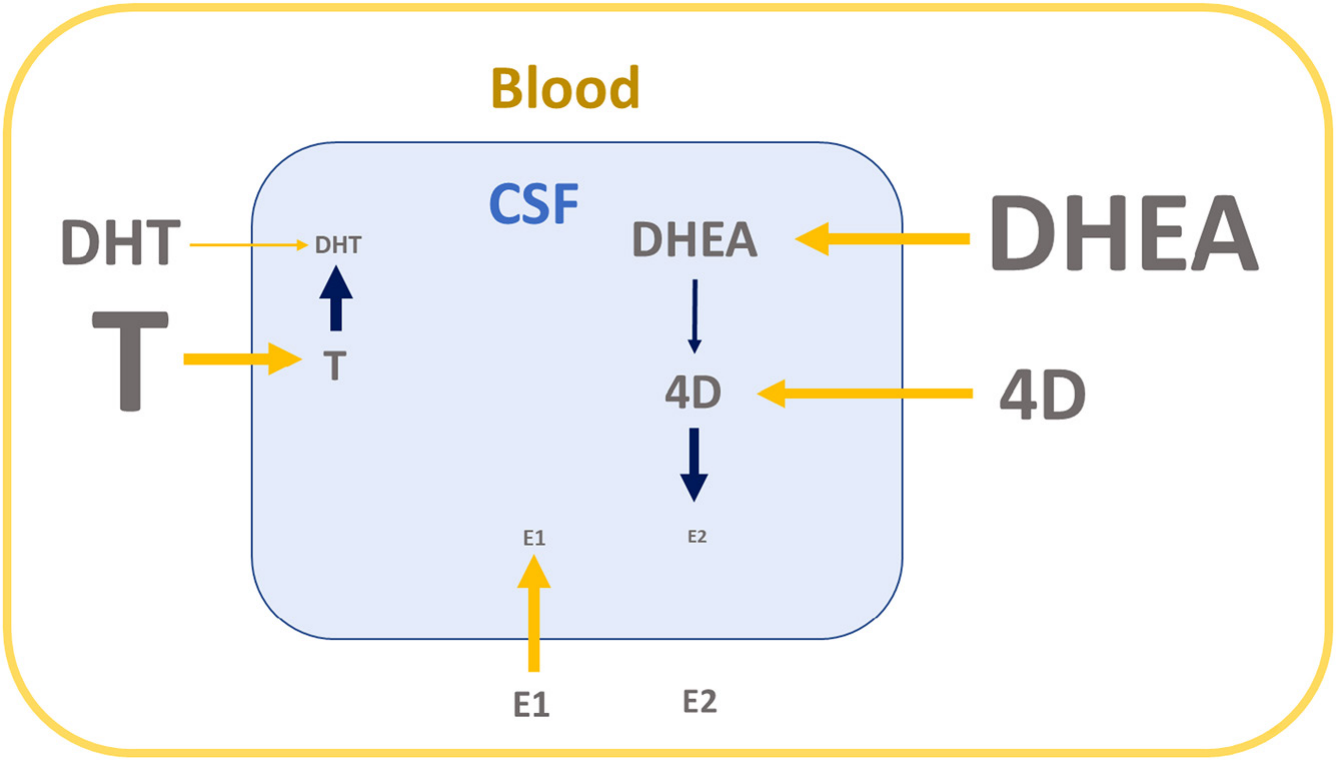
Proposed origin of sex steroids in CSF in men based on associations between sex steroids in CSF and blood. For each CSF sex steroid to be evaluated, we considered the strongest association with sex steroids in blood as well as the strongest association with other sex steroids in CSF (indicated in bold in Table 4). The relative contribution of the strongest circulating sex steroid and the strongest CSF sex steroid was determined in a combined linear regression model including both parameters (see result section). Based on the results from these analyses, we propose that testosterone in CSF is mainly derived from circulating testosterone, DHT in CSF is mainly derived from local conversion from testosterone in CSF, E2 in CSF is mainly derived from local conversion from 4D in CSF and the two sex steroid precursors (DHEA and 4D) in CSF are to a large extent derived from corresponding circulating DHEA and 4D. 4D, androstenedione; DHEA, dehydroepiandrosterone; DHT, dihydrotestosterone; E1, estrone; E2, estradiol; P, progesterone. The font sizes of the different sex steroids reflect the levels of respective sex steroid in blood and CSF. The wider the arrow the larger the estimated contribution. Yellow arrows indicate transport from blood to CSF through the BBB. Navy blue arrows indicate metabolism within CNS.

We performed measurements in 47 men with the following characteristics (mean ± s.d.): age, 72.7 ± 4.07 years; height, 1.76 ± 0.07 m; weight, 75.8 ± 10.3 kg; and BMI, 24.6 ± 2.7 kg/m^2^. The measured mean sex steroid levels in serum and CSF as well as CSF–serum ratios (CSF level/serum level × 100) are shown in Table 3. The median and range values are shown in Supplementary Table 5. In serum, testosterone (mean ± s.d.: 4365 ± 1612 pg/mL) was the major sex steroid, accounting for roughly half of the total amount of the measured sex steroids, followed by DHEA (2130 ± 1193 pg/mL). In contrast, in CSF, DHEA was the major sex steroid (73.5 ± 31.7 pg/mL), and other sex steroids found at relatively high levels in CSF were 4D (61.4 ± 29.6 pg/mL) and testosterone (49.5 ± 18.9 pg/mL), while the levels of DHT, E2, and E1 were substantially lower (Table 3 and Fig. 1). CSF levels of P were below the LLOQ for all samples except four. The calculated CSF–serum ratios showed that testosterone and DHT had the lowest ratios, around 1%, while 4D (8%) and DHEA (4%) had the highest ratios (Table 3).

**Table 3:** Sex steroid levels in serum and CSF of men (*n* = 47).

	Serum, pg/mL	CSF, pg/mL	CSF–serum ratio, %
DHT	377 ± 172	2.21 ± 0.96	0.70 ± 0.36
Testosterone	4365 ± 1612	49.5 ± 18.9	1.23 ± 0.45
E1	31.7 ± 11.0	1.02 ± 0.29	3.44 ± 1.05
E2	20.5 ± 6.25	0.62 ± 0.34	3.40 ± 2.44
DHEA	2130 ± 1193	73.5 ± 31.7	4.07 ± 1.83
4D	774 ± 259	61.4 ± 29.6	8.08 ± 2.44
P	77.0 ± 9.51	<LLOQ	–

Levels are shown as mean ± s.d. The values for testosterone are a subset of data published previously (16).

4D, androstenedione; DHEA, dehydroepiandrosterone; DHT, dihydrotestosterone; E1, estrone; E2, estradiol; P, progesterone.

Correlations between different serum sex steroid levels were as expected and are shown in Supplementary Table 2.

### Correlations between CSF and serum sex steroid levels

In Table 4, correlations between CSF and serum sex steroid levels are shown for androgens (DHT and testosterone), estrogens (E2 and E1), and sex steroid precursors (DHEA and 4D).

**Table 4:** Correlations between CSF sex steroids and serum sex steroids in men (*n* = 47).

			CSF					
			Androgens		Estrogens		Precursors	
			DHT	Testosterone	E1	E2	DHEA	4D
Serum	Androgens	DHT	**0.41^b^**	0.31^a^	−0.38^b^	−0.26	−0.04	0.05
		Testosterone	0.23	**0.48^c^**	−0.40^b^	−0.27	0.00	0.15
	Estrogens	E1	−0.09	−0.07	**0.53^c^**	0.23	0.23	0.26
		E2	0.13	0.26	−0.02	−0.1	−0.04	0.04
	Precursors	DHEA	−0.19	0.03	0.14	−0.06	**0.63^c^**	0.37^a^
		4D	−0.17	−0.05	0.29^a^	**0.33^a^**	0.42^b^	**0.62^c^**
CSF	Androgens	DHT	1	**0.52^c^**	0.00	−0.00	0.11	−0.09
		Testosterone	**0.52^c^**	1	0.04	−0.05	0.22	0.15
	Estrogens	E1	0.00	0.04	1	0.27	0.33^a^	0.28
		E2	−0.00	−0.05	0.27	1	0.02	0.39^b^
	Precursors	DHEA	0.11	0.22	**0.33^a^**	0.02	1	**0.52^c^**
		4D	−0.09	0.15	0.28	**0.39^b^**	**0.52^c^**	1

The associations are grouped for CSF androgens (DHT and testosterone), CSF estrogens (E1 and E2), and CSF sex steroid precursors (DHEA and 4D). Pearson’s correlations are given in the table. For each CSF sex hormone/precursor (i.e. for each column in the table), the strongest association with circulating sex steroids as well as the strongest association with other sex steroids in CSF is given in bold.

^a^*P* < 0.05; ^b^*P* < 0.01; ^c^*P* < 0.001.

4D, androstenedione; DHEA, dehydroepiandrosterone; DHT, dihydrotestosterone; E1, estrone; E2, estradiol; P, progesterone.

For the two sex steroid precursors, the two androgens and E1, strong positive correlations between their respective levels in CSF and serum were observed (*r* > 0.4; Table 4). In contrast, there was no correlation between E2 in CSF and E2 in serum (Table 4).

In CSF, there were strong positive correlations between the two androgens (testosterone and DHT) and between the two sex steroid precursors (DHEA and 4D). In addition, a moderate positive correlation was observed in CSF between E2 and 4D.

### Possible origin of sex steroids in the CSF

We next aimed to estimate the origin of sex steroids in CSF. Since the sex steroid levels in the circulation were consistently substantially higher than the corresponding levels in CSF (Table 3), we hypothesized that circulating sex steroids contribute to CSF levels but that local sex steroid metabolism also occurs in the CNS. For each sex steroid in CSF to be evaluated, we considered the strongest association with circulating sex steroids as well as the strongest association with other sex steroids in CSF (indicated in bold in Table 4). The relative contributions of the circulating sex steroid with the strongest association and the CSF sex steroid with the strongest association were determined in combined linear regression models including both parameters (Table 4 and Fig. 1). For DHT in CSF, we included serum DHT and CSF testosterone in the combined model (Table 4), showing that CSF testosterone was a strong (standardized *β* 0.44; *P* = 0.001) and serum DHT a modest (standardized *β* 0.27; *P* = 0.041) independent predictor of DHT in CSF (Fig. 1). For testosteronein CSF,both serum testosterone and CSF DHT were correlated with CSF testosterone, but as DHT cannot be metabolized to testosterone, serum testosterone is most likely the main source of testosterone in CSF (Fig. 1). For E1 in CSF, we included serum E1 and CSF DHEA in the combined model (Table 4), showing that serum E1 (standardized *β* 0.48; *P* < 0.001) but not CSF DHEA was independently associated with E1 in CSF (Fig. 1). In addition, we observed modest negative correlations between E1 in CSF and the two androgens in serum (Table 4).

Combined model for E2 in CSF including serum 4D and CSF 4D revealed that CSF 4D (standardized *β* 0.39; *P* < 0.007) but not serum 4D was independently associated with CSF E2. For DHEA in CSF, we included serum DHEA and CSF 4D in the combined model (Table 4), showing that serum DHEA was a strong (standardized *β* 0.51; *P* < 0.001) and CSF 4D a modest (standardized *β* 0.34; *P* = 0.005) independent predictor of DHEA in CSF (Fig. 1). Finally for 4D in CSF, we included serum 4D and CSF DHEA in the combined model (Table 4), showing that serum 4D was a strong (standardized *β* 0.48; *P* < 0.001) and CSF DHEA a modest (standardized *β* 0.32; *P* = 0.012) independent predictor of 4D in CSF (Fig. 1).

## Discussion

In the present study, we used a high-sensitive GC-MS/MS assay previously validated for sex steroid measurements in human and rodent serum (14). Here, we validated and applied this assay to determine CSF levels of DHT, testosterone, E1, E2, DHEA, 4D, and P in humans. To our knowledge, these measurements represent the most sensitive validated mass spectrometric determinations that have been presented so far of a broad panel of sex steroid hormones in human CSF.

The measured CSF sex steroid levels were in the lower pg/mL range, and for immunoassays, evidence exists of analytical interferences at these low levels (17, 18). Future research on CSF sex steroid levels should, therefore, rely on mass spectrometric assays, and there is a need to reconfirm previous data generated by immunoassays. Earlier studies using mass spectrometric assays to quantify sex steroid levels in CSF have been of limited use due to measurements of only a few sex steroids, lack of complete validation information, or insufficient sensitivity (19, 20, 21, 22).

We applied our novel assay to 47 CSF samples and 47 corresponding serum samples from healthy older men. The sex steroid levels in human serum measured using our GC-MS/MS assay are well in line with those published previously using MS, both by us using GC-MS/MS (14) and using a validated LC-MS assay (23) to establish serum reference intervals in 4678 individuals (24). In Supplementary Table 1, we compared these reference levels for testosterone, E2, 4D, and P with our measurements and found that the serum levels measured in the present study were well centered in the reference intervals, which supports the accuracy of our measurements. Finally, correlations between serum sex steroid levels have been described in previous studies, and the correlations observed in the present study are in accordance with those found earlier (25, 26).

Analyses of sex steroid levels in CSF of men in the present study revealed that DHEA was the major sex steroid followed by 4D and testosterone. The CSF levels of DHT, E2, and E1 were substantially lower, and P was in general not detectable in CSF. Previously, several studies have attempted to quantify sex steroids in CSF using MS (12, 19, 20, 21, 27, 28), but the reported levels have varied considerably. The three sex steroid hormones/precursors with the highest CSF levels (DHEA, 4D, and testosterone) have displayed the greatest consistency between studies using MS. For example, CSF 4D levels in a previous study were in average 74 pg/mL in men (20), while we in the present study found levels at an average of 62 pg/mL. For CSF testosterone in men, an average of 30 pg/mL has been reported (21) compared with 50 pg/mL in our study, but also higher levels of testosterone has been reported, 122 and 190 pg/mL (12, 22). For CSF DHEA, an average value of 100 pg/mL (21) in men has been reported, compared to 74 pg/mL in the present study. However, for sex steroid hormones found at lower levels such as DHT and E2 in men, there is little agreement between studies using MS, which might be due to lack of sensitivity, specificity, and level of validation of the previously used assays for CSF measurements. For example, using MS, Caruso *et al.* (21) suggested DHT to be around 350 pg/mL (a factor 100 above the levels measured by us), while another study reported the DHT levels in CSF of men to be undetectable (22). Thus, even mass spectrometric analyzers, capable of sensitive and specific measurements, need suitable validation to correctly measure the low levels of steroid hormones expected in CSF. Immune-based assays, on the other hand, lack the sensitivity for steroid hormone measurements in CSF, as demonstrated when applied to lower levels (but above CSF levels) of steroid hormones in serum (17). This strongly supports the use of thoroughly validated mass spectrometric assays for CSF sex steroid measurements and that immune-based assays should be avoided as they lack the required specificity and sensitivity.

In our study, the CSF–serum ratios varied considerably between different sex steroids/precursors. The androgens testosterone and DHT had the lowest ratios, at about 1%, while E1, E2, and DHEA had ratios in the midrange (3-4%), while 4D had highest ratio at about 8%. The mechanisms underlying these differences in the CSF–serum ratios are largely unknown but most likely reflect processes such as their ability to cross the blood–brain barrier, their local production and metabolism within the CNS as well as their binding affinities (highest for androgens, lowest for DHEA and 4D) for SHBG in the circulation (29).

The sex steroid levels in circulation were consistently substantially higher than corresponding levels in CSF in the present study, suggesting that circulating levels might have the capacity to influence corresponding CSF levels. This notion is supported by the strong positive correlations between their respective levels in CSF and serum observed for DHT, testosterone, E1, DHEA and 4D. In contrast, there was no correlation between E2 in CSF and E2 in serum. However, E2 in CSF was positively correlated with 4D in CSF.

We next aimed to estimate the origin of all evaluated sex steroids in CSF in men. We hypothesized that circulating sex steroids contribute to CSF levels but that local sex steroid metabolism also occurs in the CNS. For each sex steroid in CSF to be evaluated, we considered the strongest association with circulating sex steroids as well as the strongest association with other sex steroids in CSF. The relative contributions of the circulating sex steroid with the strongest association and the CSF sex steroid with the strongest association were determined in combined linear regression models including both parameters. Based on the results from these analyses, we propose that testosterone in CSF is mainly derived from circulating testosterone, DHT in CSF is mainly derived from local conversion from testosterone in CSF, E2 in CSF is mainly derived from local conversion from 4D in CSF and the two sex steroid precursors (DHEA and 4D) in CSF are to a large extent derived from corresponding circulating DHEA and 4D (Fig. 1). However, based on known steroidogenic pathways, we cannot exclude the possibility that the conversion of 4D to E2 in CSF could also involve intermediary steroids that were not identified in our statistical analysis. Although these potential intermediary steroids are yet to be identified, possible candidates could include testosterone (D4 to testosterone to E2) or E1 (D4 to E1 to E2). Collectively, our proposed model indicate that testicular-derived androgens enter the CNS as testosterone, which is then locally metabolized to DHT in men. In addition, adrenal-derived sex steroid precursors easily enter the CNS and can be metabolized locally into E2 (Fig. 1). It should be emphasized that these models are based on cross-sectional associations and that functional studies are warranted to validate the proposed models (Fig. 1).

Interestingly, we observed that the serum levels of both testosterone and DHT were inversely associated with E1 in CSF. This is in some accordance with a previously described estrogen-dependent negative feedback of androgen production through a gonadotropin-dependent mechanism (30). In the present study, we used a comprehensive panel of sex steroid hormones and precursors. However, conjugated steroids were not included as these are not possible to measure directly using our assay. Moreover, androgen and progestin metabolites/precursors such as androsterone, 11-oxygenated androgens, and 11-keto-androgens would also have been of interest (31). Thus, future studies with broader panels could generate a more precise model over the contributions of peripheral steroids and local production to the steroid levels in CSF. Finally, we studied older men and steroid levels could be different in other groups. For example, higher levels of E1 and E2 together with lower levels of testosterone and DHT in CSF are expected in premenopausal women, while low levels of both estrogens and androgens are expected in postmenopausal women and children. Future studies including females as well as younger individuals are therefore needed.

Another limitation lies in the number of aliquots used. Difficulties in obtaining large quantities of CSF with low endogenous steroid levels limited the number of aliquots used (*n* = 5) at each level. However, the total number of analyses were quite high, as seven different levels were analyzed.

In summary, we have validated a highly sensitive and specific GC-MS/MS method for analyses of a broad panel of sex steroids in human CSF. Using this method, we demonstrate that DHEA is the major sex steroid followed by 4D and testosterone, while the levels of DHT, E2, and E1 are substantially lower in CSF in men. In addition, we propose a tentative model of possible pathways for sex steroid transportation into the CNS and conversion of sex steroids within the CNS. The presented new assay for analyses of a comprehensive sex steroid profile in human CSF constitutes a valuable tool for investigation of the role of sex steroids in the human CNS, both in healthy subjects and in disease states.

## Supplementary Materials

Supplementary Material

## Declaration of interest

‘The authors declare that there is no conflict of interest that could be perceived as prejudicing the impartiality of the study reported.

## Funding

Claes Ohlsson: Swedish Research Council, the Swedish state under the agreement between Swedish government and county councils, the ALF-agreement ALFGBG, Lundberg Foundation for Research and Education, Torsten Söderberg Foundation, Novo Nordiskhttp://dx.doi.org/10.13039/501100004191 Fonden, Knut and Alice Wallenberg Foundation.
