# Supplementary material for: Sex steroid levels in corresponding cerebrospinal fluid and serum samples quantified by mass spectrometry in men
Source: Endocr Connect. 2023 Dec 14;13(1):e230250. doi: 10.1530/EC-23-0250 (PMC10762557; doi:10.1530/EC-23-0250)
Supplement: Supplementary Material [file supplementary_material.pdf]

Supplementals

	Present study	*Bea et al*<br>60-80 years
***T** (pg/ml)*	4370±1610	1834-7585
***E2** (pg/ml)*	20.5±6.25	<44.4
***4D** (pg/ml)*	774±259	263-1366
***P** (pg/ml)*	77.0±9.51	<223

Supplemental Table 1. Comparison between serum sex steroid measurements in the present study and reference values in men published by Bae *et al* recalculated to pg/mL (1). T stands for testosterone; E2, estradiol; 4D, androstenedione and P, progesterone.

	DHT	***T***	***E1***	***E2***	***DHEA***	***4D***
DHT						
T	0.84***					
E1	0.02	0.04				
E2	0.51***	0.58***	0.49***			
DHEA	-0.04	0.06	0.19	-0.08		
4D	0.04	0.13	0.39**	0.07	0.58***	
P	-0.06	-0.08	-0.07	0.00	-0.06	-0.02

Supplemental Table 2. Pearson's correlations between serum steroids in men (n=47). DHT stands for dihydrotestosterone, T; testosterone, E1; estrone, E2; estradiol, DHEA; dehydroepiandrosterone, 4D; androstenedione and P; progesterone. * $p< 0.05$; ** $p< 0.01$; *** $p< 0.001$

	Calibrator stocks (µg/mL)	***Level 1 (pg/mL)***	***Level 2 (pg/mL)***	***Level 3 (pg/mL)***	***Level 4 (pg/mL)***	***Level 5 (pg/mL)***	***Level 6 (pg/mL)***	***Level 7 (pg/mL)***	***R2***
E2	80	800	120	16	8	2	1	0.5	>0.999
E1	80	800	120	16	8	2	1	0.5	>0.999
DHT	200	2 000	300	40	20	5	2.5	1.25	>0.999
4D	500	5 000	750	100	50	12.5	x	x	>0.999
T	500	10 000	1 500	200	100	25	12.5	6.25	>0.999
DHEA	200	20 000	3 000	400	200	50	25	x	>0.999
P	200	20 000	3 000	400	200	50	25	12.5	>0.999

Supplemental Table 3. Calibrator stocks, calibrator dilutions and calibrator standards used in CSF measurements. For serum measurements, levels are multiplied with the factor 1.8 as 250 µL was used for serum measurements and 450 µL for CSF measurements.

Seven different calibrator stocks, one for each steroid hormone, were made in ethyl acetate. The calibrator stocks were used to make seven calibrator dilutions (1:100), one for each steroid hormone. The seven different calibrator dilutions were pooled and diluted with methanol 1:100 to achieve calibrator level 1. Level 2 was 7.5:50 of level 1, level 3 was 1:50 of level 1, level 4 was 1:100 of level 1, level 5 was 0.835:50 of level 2, level 6 was 0.835:100 of level 2, and level 7 was 0.835:200 of level 2. On the day of analysis, calibrator samples were made by spiking water with 45µL of each calibrator level. The calibrator concentrations for CSF measurements are shown in the table are after this final 1:10 dilution step (45:450).

For some steroids, the lowest calibrator points were not used as they were below the LLOQ, denoted with x in the table.

All calibrator stocks, dilutions and solutions were stored at -80°C and will be stable for at least 5 years at this temperature. Between experiments, calibrator levels were kept at +4°C, normally for maximal four weeks. Calibrator levels are stable for up to 2 months at +4°C.

DHT stands for dihydrotestosterone, T; testosterone, E1; estrone, E2; estradiol, DHEA; dehydroepiandrosterone, 4D; androstenedione and P; progesterone. R2 is the linear correlation coefficient for a representative calibration curve.

Internal standard	***Concentration (pg/mL)***
DHT-$^{13}C_3$	400
T-$^{13}C_3$	20 000
E1-$^{13}C_3$	400
E2-$^{13}C_3$	800
DHEA-D_6	20 000
4D-$^{13}C_3$	10 000
P-$^{13}C_3$	100 000

Supplemental Table 4. Concentrations of internal standards in spike solution. Prior sample preparation, 50µL of the solution was spiked to the sample. DHT stands for dihydrotestosterone, T; testosterone, E1; estrone, E2; estradiol, DHEA; dehydroepiandrosterone, 4D; androstenedione and P; progesterone.

	Serum<br>*pg/mL*	***CSF***<br>*pg/mL*
DHT	373 (101-780)	1.61 (1.50-5.30)
T	4464 (1315-7545)	45.7 (14.0-104)
E1	31.2 (14.3-74.0)	0.88 (0.88-1.83)
E2	18.7 (9.44-35.9)	0.59 (0.25-1.88)
DHEA	2868 (550-5300)	67.6 (41.2-163.2)
4D	732 (368-1440)	55.2 (23.4-210)
P	74 (74-134)	<LLOQ

Supplemental Table 5: Median sex steroid levels in serum and CSF of men (n=47). Levels are shown as medians (range). DHT stands for dihydrotestosterone, T; testosterone, E1; estrone, E2; estradiol, DHEA; dehydroepiandrosterone, 4D; androstenedione and P; progesterone.

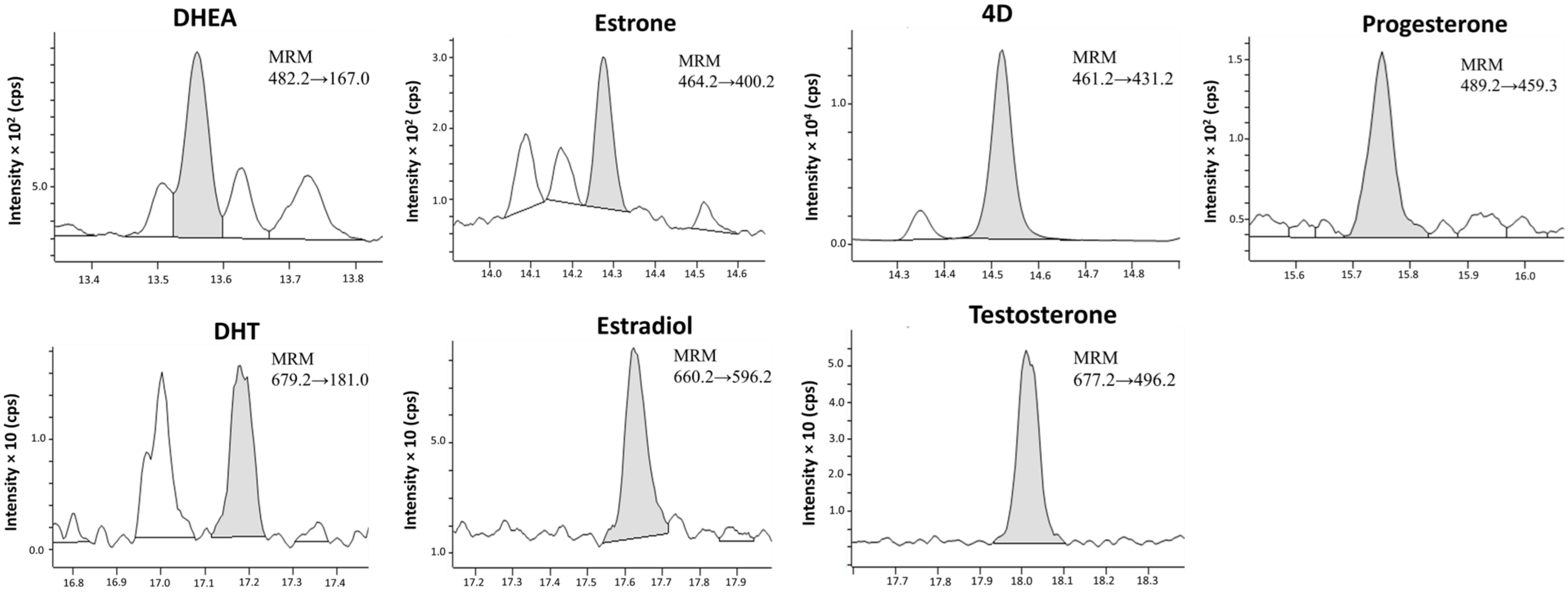

Supplement Figure 1. Representative extracted ion chromatograms in CSF for following steroids and their concentrations: Dehydroepiandrosterone (DHEA), 147 pg/mL; estrone (E1), 2.6 pg/mL; androstenedione (4D), 122 pg/mL; progesterone, 4.5 pg/mL; dihydrotestosterone (DHT), 7.0 pg/mL; estradiol (E2), 1,7 pg/mL; testosterone (T), 58 pg/mL.

References

1. Bae YJ, Zeidler R, Baber R, Vogel M, Wirkner K, Loeffler M, Ceglarek U, Kiess W, Korner A, Thiery J, Kratzsch J. Reference intervals of nine steroid hormones over the life-span analyzed by LC-MS/MS: Effect of age, gender, puberty, and oral contraceptives. *The Journal of steroid biochemistry and molecular biology*. 2019;193:105409.
